# Stress Evaluation in Adult Patients with Atopic Dermatitis Using Salivary Cortisol

**DOI:** 10.1155/2013/138027

**Published:** 2013-07-23

**Authors:** Megumi Mizawa, Masaki Yamaguchi, Chieko Ueda, Teruhiko Makino, Tadamichi Shimizu

**Affiliations:** ^1^Department of Dermatology, Graduate School of Medicine and Pharmaceutical Sciences, University of Toyama, 2630 Sugitani, Toyama-shi, Toyama 930-0194, Japan; ^2^Graduate School of Engineering, Iwate University, 4-3-5 Ueda, Morioka-shi, Iwate 020-8551, Japan

## Abstract

The symptoms of atopic dermatitis (AD) are often aggravated by stress, and AD can also lead to psychological stress due to social isolation and discrimination. The salivary cortisol level reflects psychological stress, and it is a good index to assess chronic stress. In this study, we measured the salivary cortisol levels in patients with AD (*n* = 30) and compared them with those of healthy control subjects (*n* = 42). AD patients were also evaluated for general disease severity using the Scoring Atopic Dermatitis (SCORAD) index. The serum levels of TARC, total IgE, and lactate dehydrogenase (LDH) and peripheral blood eosinophil counts were measured by laboratory tests. The Skindex-16 was used as a skin disease-specific, quality of life measure, instrument. The results showed that the saliva cortisol level was significantly higher in AD patients compared to healthy subjects (*P* < 0.01). The salivary cortisol level was significantly correlated with the SCORAD index (*r* = 0.42, *P* < 0.05) while the serum TARC and LDH levels were positively correlated with the SCORAD index. However, no statistically significant correlations were observed between the salivary cortisol level and Skindex-16. These results suggest that the saliva cortisol level is therefore a useful biomarker to evaluate the stress in AD patients.

## 1. Introduction

Atopic dermatitis (AD) is a common chronic inflammatory skin disease characterized by inflammatory infiltration, extensive pruritus, and a clinical course defined by symptomatic flares and remissions. The pathogenesis of the disease is becoming better understood, and important clues about the pathogenesis of the disease have been discovered, including genetic factors, skin barrier dysfunction, and immune dysregulation [1]. AD can lead to psychological stress, due to stigmatization, social isolation, and discrimination. It has been reported that the stress after the Hanshin Awaji earthquake disaster in Japan influenced the symptoms of AD, suggesting that natural disasters affect AD symptoms [2]. Furthermore, exacerbation of AD symptoms has been seen in patients under the influence of stress [3, 4], thus implying the fact that emotional factors contribute to the severity of AD. 

The evaluation of stress biomarker would be helpful to control skin inflammation by allowing for a more proactive management of AD patients. However, so far, no reliable biomarkers are available to assess the level of stress in AD patients. The salivary cortisol level is known to be a psychological stressor and is a useful index to assess chronic stress [5]. Additionally, saliva sampling has the advantage of being noninvasive, making multiple sampling easy and stress-free.

In this study, we examined the salivary cortisol levels in patients with AD and compared them with those of healthy control subjects.

## 2. Materials and Methods

### 2.1. Subjects

The study subjects included thirty AD patients (15 males and 15 females; age, 15–62 years; mean age, 29.6 years). The diagnosis of AD was based on the Rajka and Hanifin criteria, and patients had no other concomitant diseases. Forty-two normal healthy control subjects (27 males and 15 females; age, 31–54 years; mean age, 39.4 years) were also enrolled in this study. The study was approved by the Ethics Committee of the University of Toyama and Iwate University.

### 2.2. Collection of Saliva

Experimental sessions were limited to the hours between 9:00 and 11:00 a.m. to minimize time of day effects. Prior to the experiment, the subjects rinsed their mouth in order to clean the oral cavity and then waited five minutes. A braided cotton dental rope was then placed in their mouth and left in place for five minutes to collect saliva. Saliva samples were collected from AD patients and normal healthy controls. Then, the saliva samples were centrifuged, and the supernatant was stored at −80°C until it was analyzed.

### 2.3. Salivary Cortisol Analysis

The salivary cortisol levels were measured using commercial linked immunosorbent assay kits (1-3002; Salimetrics LLC, State College, PA) and a plate reader (450 nm measurement wavelength; ARVO MX; Perkin Elmer Life Science, Boston, MA). The intra- and interassay variations were 3.35-3.36 and 3.75–6.41%, respectively. The cortisol concentration was expressed in nanomoles per liter.

### 2.4. Assessment of Clinical Severity

AD patients were also evaluated for the general disease severity using the Scoring Atopic Dermatitis (SCORAD) index. The serum thymus and activation-regulated chemokine (TARC) level, serum total IgE level, serum lactate dehydrogenase (LDH) level, and peripheral blood eosinophil counts were measured by standard laboratory tests.

### 2.5. Skin Disease-Specific Quality of Life (QOL) Assessment

The Japanese version of the Skindex-16, consisting of 16 items in three scales (symptoms, emotions, and functioning), was used as a skin disease-specific instrument. In the present study, the subjects described the degree of anguish caused by the disease by assessing each item on a scale of 0 (never) to 6 (all of the time). Each of the 16 items was quantified using a scale from 0 to 100, and the average was calculated.

### 2.6. Statistical Analysis

Data are presented as the mean values and standard errors of the means (±S.E). Mann-Whitney's *U* test was used to assess the salivary parameters between the test and control groups. Pearson's correlation coefficient (*r*) was determined for the relationship between the SCORAD index and levels of salivary cortisol. The correlation between levels of salivary cortisol and AD-related clinical severity markers and Skindex-16 were performed using Pearson's correlation coefficient while IgE and LDH were analyzed by Spearman's rank correlation coefficient. Statistical significance value was accepted at *P* < 0.05.

## 3. Results

### 3.1. Clinical Characteristics

The distribution of the SCORAD index in the enrolled AD patients ranged from 9.9 to 80.3 (46.7 ± 3.2, mean ± SE). The results of laboratory tests are summarized in Table 1. The SCORAD indices were positively correlated with both the serum TARC levels (*r* = 0.57, *P* < 0.01) and the serum LDH levels (*r* = 0.46, *P* < 0.05). However, no statistically significant correlation was observed between the SCORAD indices and serum IgE levels (*r* = 0.30, *P* = 0.12) or the number of peripheral blood eosinophils (*r* = 0.27, *P* = 0.16). The Skindex-16 was measured as an assessment of skin disease-specific quality of life (QOL). The emotional domain was the most affected domain, followed by the symptoms and functioning domains. However, no statistically significant correlations were observed between the SCORAD indices and the Skindex-16 (*r* = 0.19, *P* = 0.32).

### 3.2. Salivary Cortisol Levels

**Figure 1:**
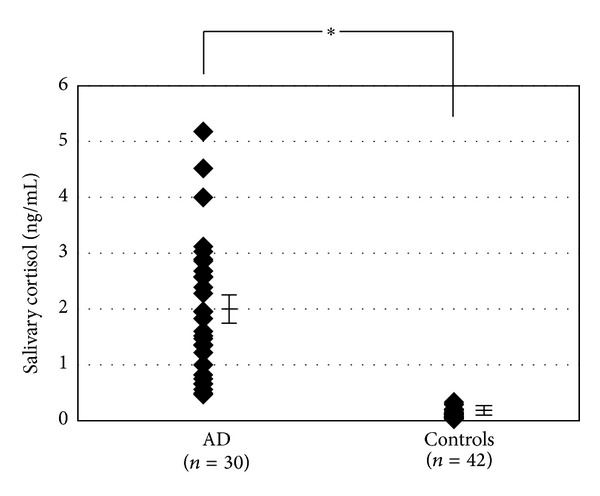
Comparison of the salivary cortisol levels in AD patients and normal healthy controls. The salivary cortisol levels (ng/mL) in AD patients (*n* = 30) were compared with those in controls (*n* = 42). *P < 0.01.

**Figure 2:**
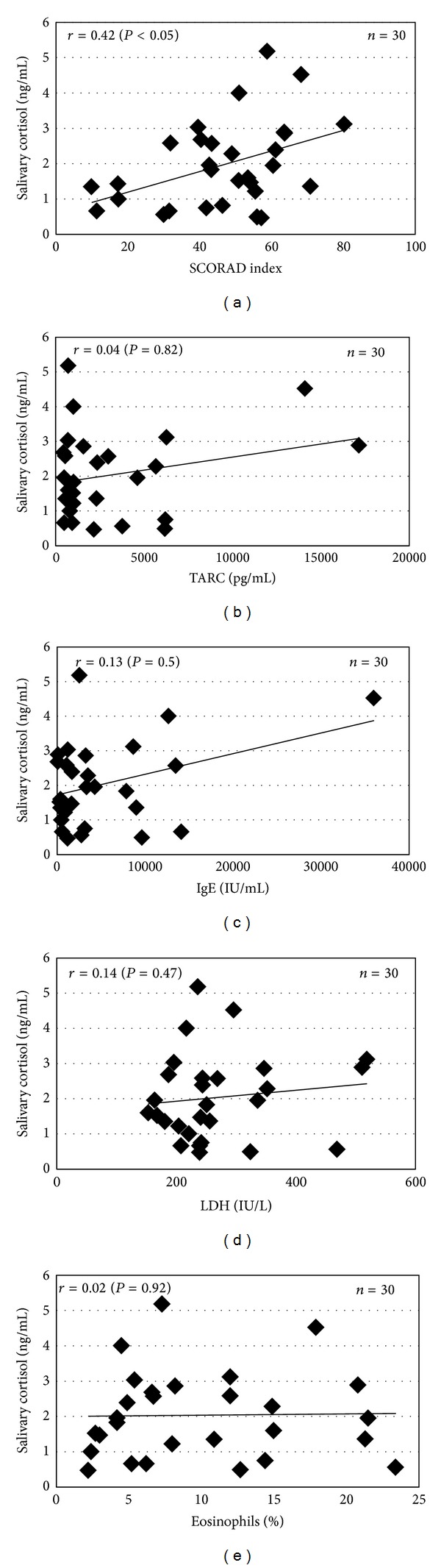
Correlation between the salivary cortisol levels, the SCORAD index, and other clinical severity markers in AD patients. (a) Significant correlation between the SCORAD index and salivary cortisol levels (ng/mL) (*r* = 0.42, *P* < 0.05). (b–e) No significant correlations were observed between the salivary cortisol level and the serum levels of TARC, IgE, and LDH or the number of peripheral blood eosinophils.

**Figure 3:**
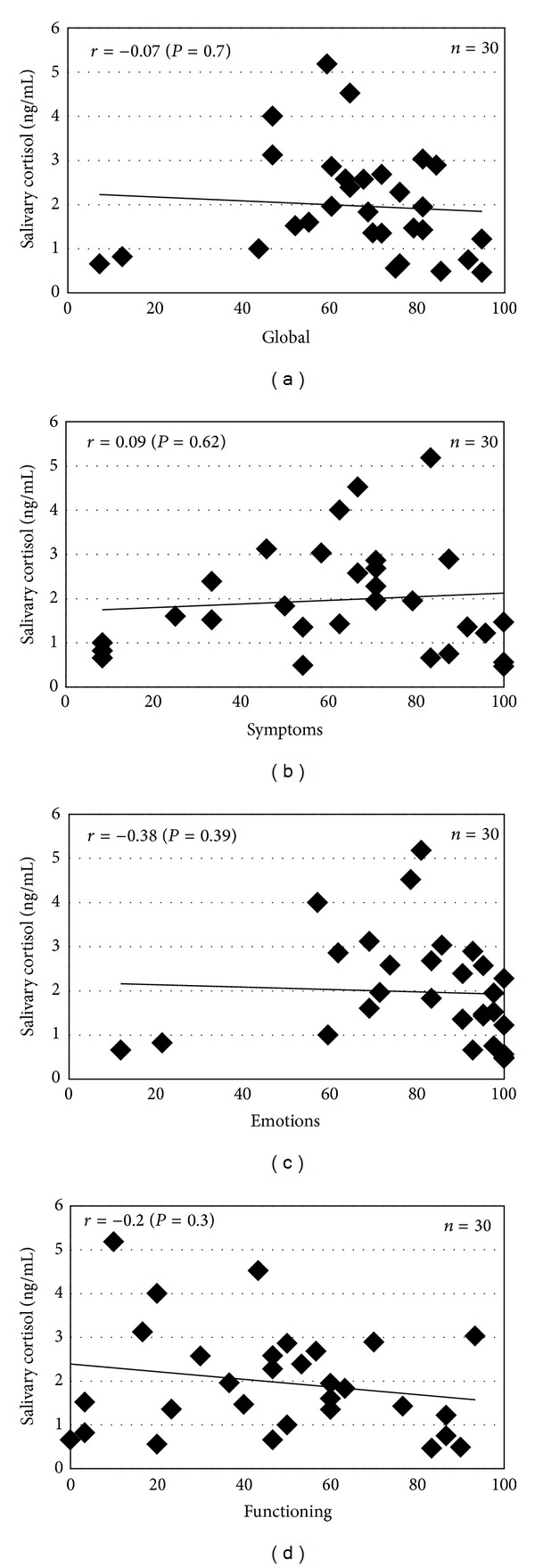
Correlation between the salivary cortisol levels and the Skindex-16 in AD patients. (a–d) No statistically significant correlations were observed between the salivary cortisol levels and Skindex-16 (global, symptoms, emotions, or functioning).

We examined the salivary cortisol levels in patients with AD and compared them with those of healthy control subjects. The salivary cortisol level in AD patients ranged from 0.47 to 5.18 ng/mL (1.97 ± 0.22 ng/mL; mean ± SE), which was significantly higher compared to that of the healthy controls (from 0.028 to 0.334 ng/mL; 0.11 ± 0.01 ng/mL; *P* < 0.01) (Figure 1). We next analyzed the relationship between the salivary cortisol levels and other clinical severity markers and the Skindex-16. The levels of salivary cortisol were significantly correlated with the SCORAD index (*r* = 0.42, *P* < 0.05) (Figure 2(a)). However, the serum levels of TARC, IgE, and LDH or the number of peripheral blood eosinophils did not show statistically significant correlations with the salivary cortisol level (TARC, *r* = 0.04, *P* = 0.82; IgE, *r* = 0.13, *P* = 0.50; LDH, *r* = 0.14, *P* = 0.47; eosinophils, *r* = 0.02, *P* = 0.92) (Figures 2(b)–2(e)). The correlation between salivary cortisol levels and skindex-16 was also found statistically insignificant (global, *r* = −0.07, *P* = 0.70; symptoms, *r* = 0.09, *P* = 0.62; emotions, *r* = −0.38, *P* = 0.39; functioning, *r* = −0.20, *P* = 0.30) (Figure 3).

## 4. Discussion

AD is a stress-prone disorder that involves the autonomic nervous system. Several triggers of AD have been identified such as food and airborne allergens, contact allergens, skin microorganisms, irritants, and psychological stress. The strength of the stress depends on the individual perception, subjective rating, and the extent of the stressful event. However, the actual effect of stress on AD is poorly understood due to the lack of a method to objectively quantify stress.

In this study, it was observed that the salivary cortisol level was significantly increased in AD patients in comparison to healthy subjects (*P* < 0.01). This suggests that AD patients might be suffering from chronic stress. The hypothalamic-pituitary-adrenal (HPA) axis and the sympathetic nervous system constitute the main effector pathways of the stress system. Cortisol is secreted from the adrenal cortex in the HPA axis, and its level increases not only due to acute stress, but also due to chronic stress, such as that resulting from stigmatization, social isolation, and discrimination [6–8]. Rai et al. found that stress and a salivary stress marker, cortisol, were significantly correlated with the clinical parameters of periodontal disease and suggested that stress might be associated with the activity of periodontal diseases as a result of physiological and behavioral mechanisms [9]. In a study by Koray et al., the salivary cortisol level was significantly increased in oral lichen planus patients, who often related the onset and aggravation of oral symptoms to increased levels of stress [10]. Based on these findings, the level of salivary cortisol is considered to be a useful index of chronic stress.

The present study showed that the levels of salivary cortisol were significantly correlated with the SCORAD index, which is the useful marker to determine the clinical severity of AD. Therefore, we speculated that severe AD might be associated with more stress than mild and moderate AD. Furthermore, it was considered that stress may interact with an immune pathway by acting on the central nervous system and thereby affecting the endocrine system [11]. Stress causes a decrease in the serum dehydroepiandrosterone level, affecting the Th1-type cytokine responses, thereby facilitating a shift to a Th2-type cytokine profile and exacerbating the AD symptoms. In addition, stress induces the release of substance P (SP) from C-fibers. SP activates both keratinocytes [12] and mast cells, and these activated cells synthesize and secrete more than 50 biologically active molecules, including cytokines, nerve growth factor, and histamine, which are mediators of neurogenic inflammation [13]. In contrast, no significant correlations were observed between the salivary cortisol level and other serum biomarkers, including the levels of TARC, IgE, and LDH and eosinophils. This difference might be caused by the change in the serum marker levels due to treatment with either topical corticosteroid or antihistamine. Assessment of the QOL by the Skindex-16 showed that AD patients were especially impacted with regard to the emotional domain. This might induce psychological stress in AD patients; however, no statistically significant correlations were observed between the salivary cortisol level and Skindex-16.

It was reported that the serum cortisol levels of inpatients with severe AD were significantly decreased compared to those of outpatients with mild and moderate AD [14, 15]. This finding is not inconsistent with present findings; this may be due to the fact that in the previous study it was described that approximately 88% of severe AD patients with low serum cortisol levels had sleep disorders, which is suggested to induce suppression of the endocrine system [15]. Therefore, the levels of serum cortisol might be suppressed in severe AD patients. In contrast, all of the patients we examined in this study were outpatients, and they did not have any sleep disorders. We speculate that an existence of sleep disorders may have led to the discrepancy in the cortisol levels of severe AD patients, although other factors may also be related to the cortisol level.

In conclusion, our study suggests that AD patients might be under chronic stress, and the severity of AD may be correlated with the intensity of the stress. Saliva sampling has the advantage of being noninvasive, making multiple sampling easy and stress-free. Therefore, these results suggest that the saliva cortisol level is a useful biomarker to evaluate the stress in AD patients and to help physicians in order to plan more effective treatment strategies for these patients.

## Figures and Tables

**Table 1:** Clinical characteristics and laboratory tests.

	Range	Mean ± SE
SCORAD index	9.9–80.3	46.7 ± 3.2
TARC (pg/mL)	443–17160	3093.5 ± 767.7
IgE (IU/mL)	105–36009	5180.6 ± 1404.3
LDH (IU/L)	153–519	268.4 ± 18.4
EOS (%)	2.2–23.4	9.95 ± 1.2
Skindex-16		
Global	7.3–94.8	66.3 ± 3.8
Symptoms	8.3–100	63.2 ± 5.0
Emotions	11.9–100	81.4 ± 4.0
Functioning	0–99.3	47.5 ± 5.0

EOS: peripheral blood eosinophils.

## Abbreviations

AD: Atopic dermatitis
HPA: Hypothalamic-pituitary-adrenal
LDH: Lactate dehydrogenase
QOL: Quality of life
SP: Substance P
SCORAD: Scoring atopic dermatitis
TARC: Thymus and activation-regulated chemokine.
